# Enhancing Masculine Features After Massive Weight Loss

**DOI:** 10.1007/s00266-016-0617-x

**Published:** 2016-02-18

**Authors:** Dennis Hurwitz

**Affiliations:** University of Pittsburgh Medical Center, 3109 Forbes Avenue, Pittsburgh, PA 15213 USA

**Keywords:** Male body contouring, Gynecomastia correction, Body contouring surgery, Abdominoplasty, Boomerang correction of gynecomastia, Liposuction, Upper body lift, Lower body lift

## Abstract

**Background:**

Whereas body contouring surgery after massive weight loss in women emphasizes sculptured adipose and broader lower torso, little attention has been devoted to accentuating the male physique.

**Objective:**

To determine if boomerang excision pattern correction of gynecomastia with J torsoplasty combined with an abdominoplasty with oblique excisions directly over bulging flanks provide effective and safe optimizing of muscle visibility and upper torso dominance.

**Methods:**

A description of comprehensive body contouring through an abdominoplasty and a series of obliquely oriented ellipses of the male torso is followed by review of 19 consecutive patients.

**Results:**

Seventeen patients were performed in a single stage. Nine of the last ten cases included J torsoplasty and oblique excision extensions over the flanks. Of the 17 patients responding to a ten-question survey, 15 were satisfied with chest improvement. One of the first eight patients with a transverse lower body lift was satisfied with the flank bulges. All of the last eight cases with direct oblique flank excisions were satisfied with their lower body. Five patients (26 %), having a total of 74 operative procedures, had significant complications of chest hematoma, persistent hip and buttock seromas, superior NAC edge necrosis, and distal necrosis of the fleur de lis abdominoplasty. One boomerang correction underwent minor revisions. One transverse lower body lift underwent major revision. No complications occurred in the last ten patients, having oblique flank excisions instead of transverse lower body lifts.

**Conclusion:**

Comprehensive excisional body contouring surgery of a central high tension abdominoplasty with a series of obliquely oriented ellipses throughout the torso appears to provide low risk improved body contour for the muscular male.

**Level of Evidence IV:**

This journal requires that authors assign a level of evidence to each article. For a full description of these Evidence-Based Medicine ratings, please refer to the Table of Contents or the online Instructions to Authors www.springer.com/00266.

## Introduction

After massive weight loss (MWL), men seek body contouring surgery for the removal of excess skin and fat followed by tightening and suspension of residual lax tissues. Men generally have correction of pseudogynecomastia and an abdominoplasty extending into a lower body lift [1].

**Fig. 1:**
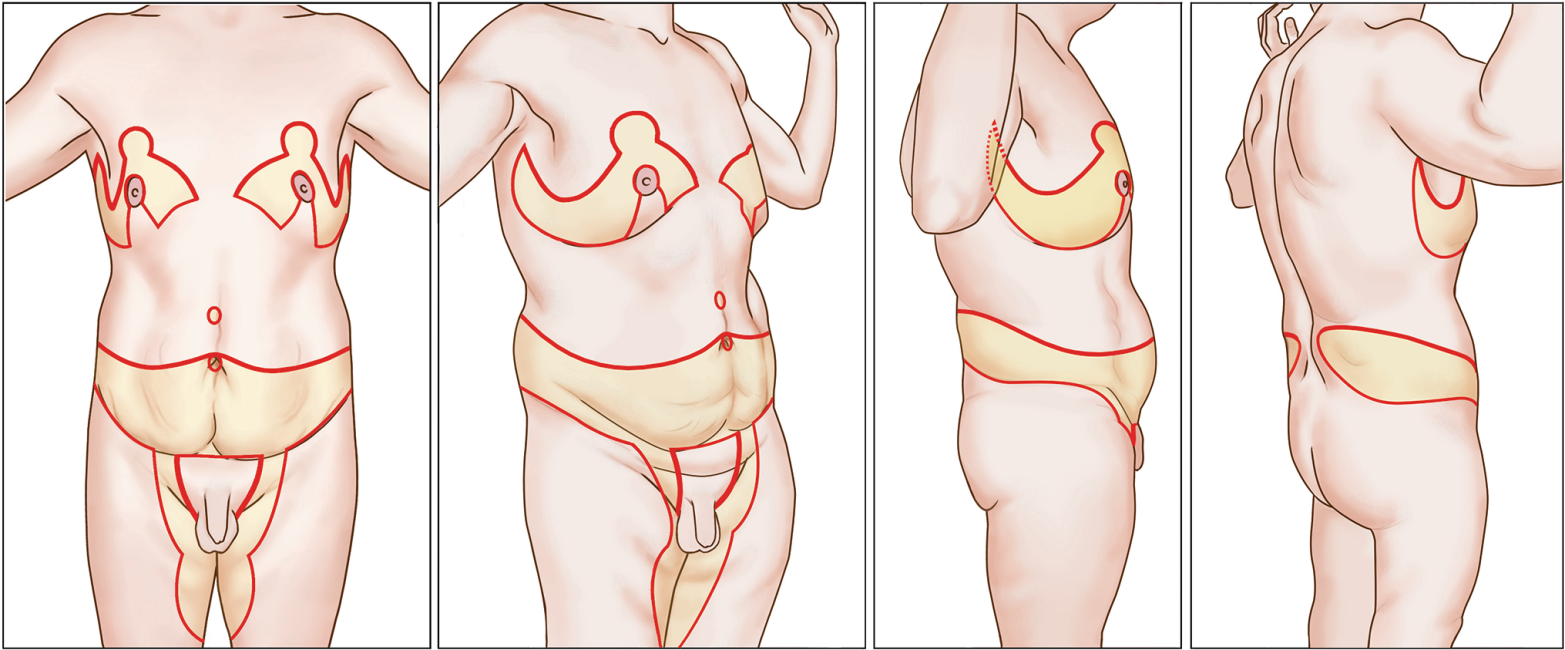
Frontal and right side artist rendering of male TBL consisting of boomerang pattern correction of gynecomastia, J torsoplasty upper body lift, abdominoplasty extended with elliptical oblique excisions of bulging flanks, and a picture frame monsplasty continuation of a vertical medial thighplasty. *Red lines* indicate incisions. *Yellow fill* represents full thickness skin and subcutaneous tissue resections

**Fig. 2:**
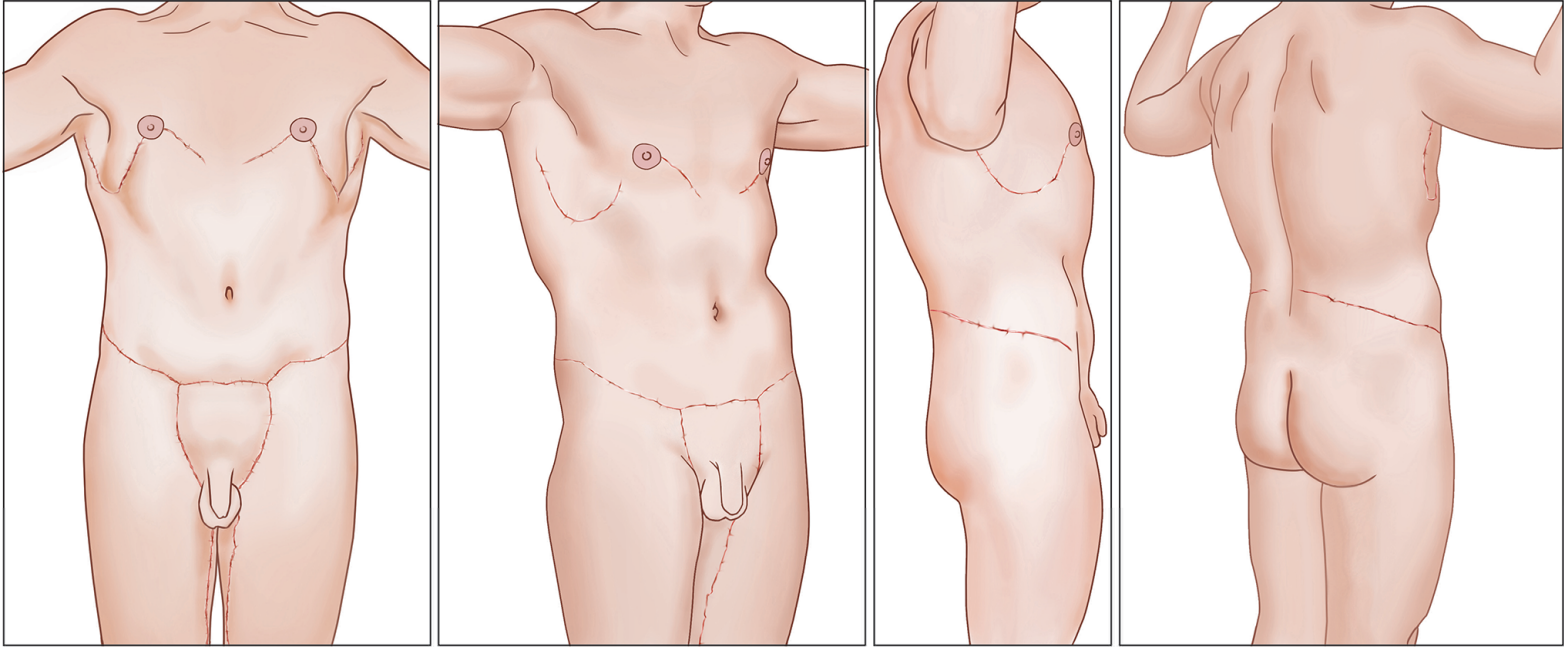
Frontal and right side artist drawings of ideal muscular male after TBL surgery. *Faint lines* indicate unobtrusive curvilinear scars. The upper torso dominates with skin tightly wrapping about superficial muscles. Defined pectoralis and latissimus dorsi muscles drape from broad shoulders. With arms slightly elevated, the pectoralis muscle is stretched and thinned, completely emptying fullness deep and inferior to nipples. Raised arms reveal the recess of intercostal and serratus muscles rippling between the prominent lateral borders of the pectoralis and latissimus dorsi muscles. The inferior and lateral borders of the pectoralis muscles are defined about the fourth rib. A flat rectus abdominus muscle, depressed by two transverse inscription sets, extends from the costal margin to the mons pubis. The rectus muscles are further contoured by narrow depressions along the midline linea alba and obliquely oriented lateral rectus boarder. As the narrow waist approaches the hip, the external oblique muscle smoothly swells above the prominent iliac crest. The lateral border of the latissimus dorsi muscle extends to nearly the hips. The buttocks are *rounded and narrow*

Since many men are obsessed with muscle show and upper torso dominance and are considering plastic surgery to achieve those goals [2], plastic surgeons should be prepared to accentuate those features. This is a preliminary report of total body lift (TBL) [3] surgery that seeks that transformation through abdominoplasty and a crisscross pattern of elliptical excisions across the torso (Fig. 1) [4]. By removal of most horizontal and vertical excess skin and fat, uniformly tight skin across the torso leaves upper body dominance, muscular show, and two sets of long zigzag scars (Fig. 2). This comprehensive surgery is presented and then followed by a review of 19 consecutive patients (Table 1).

**Table 1:** Patient data

Pt	Age	BMI	Delta BMI	Upper	Abdomen	Lower	Other operations	F/U year	Complication	Comments
1	48	33.4	7	Transverse	Yes	Transverse		8	None	
2	44	26.6	13	None	Yes	Transverse		3.3	None	Post op.30# gain,
3	47	37	10	None	Yes	Transverse	Thighplasty	0.4	Seroma, hip drain	Prolonged care
4	45	30	15.3	Transverse	FDL	Transverse		0.9	abd. wound	Secondary closure
5	50	34.4	14.6	Transverse	Yes	Oblique		5.7	Partial necr. NAC	Healed secondarily
6	23	32.5	13.4	Transverse	Yes	Transverse	Adipose buttock flap, thighplasty	10.1	Chest hematoma	Evacuated in OR
7	26	27.9	15.3	Transverse	Yes	Transverse	Adipose buttock flap	0.5	Buttock seroma	Decorticate buried flap
8	35	26.5	13	Transverse	Yes	Transverse	Thighplasty	5.8	None	
9	23	22.7	16.5	J torsoplasty	Yes	Transverse		2.6	None	NAC’s lowered
10	38	28.2	17.8	J torsoplasty	Yes	Oblique	Thighplasty, brachioplasty	1.5	None	Secondary liposuction
11	30	28.2	8.4	J torsoplasty	Yes	Oblique	Thighplasty	2.2	None	
12	31	29	15	J torsoplasty	Revision	Oblique	Repair neuroma, UAL epig.	3.3	None	Prior TBL surgery
13	27	29.2	17	J Torsoplasty	Yes	Oblique	Thighplasty	3.7	None	Post op.10# gain,
14	26	23	25.8	J torsoplasty	Yes	Oblique	Thighplasty	2.9	None	Post op. 90# gain
15	40	31	2	J torsoplasty	Revision	Oblique	Prior UAL flanks, abd. Lower	1.1	None	Prior TBL surgery
16	66	25.8	7.7	J torsoplasty	Yes	Oblique	Vertical sternal skin excision	2.8	None	Pectus excav. treated
17	24	27.8	12.8	J torsoplasty	Yes	Oblique		1.8	None	Thick medial scars
18	28	24.5	10.2	J torsoplasty	Yes (Stg 1)	Oblique	Stg 1 UAL Breasts	1.8	None	Two stages
19	42	23	16.5	J torsoplasty	Yes (Stg 1)	Transverse	Stg 1 UAL Breasts	1.6	None	Two stages
Av	36.5	28.5	13.2					3.1		
Rg	23–66	23–34	26-Feb							

Patients (1–19) are listed in the order of treatment. All patients had boomerang pattern correction of gynecomastia with and abdominoplasty extended by either a transverse or oblique lower body lift

The indication for this male TBL is sagging pseudogynecomastia with moderate to severe skin laxity of the abdomen and flanks 1 year after stable MWL. The ideal patient is muscular, healthy, and frustrated that rigorous bodybuilding fails to reveal visible results. He desires a harmonious muscularity throughout the torso with the upper dominating the lower. Less sinewy and older men, seeking more muscular show, are also considered. Lengthy operations and scars throughout their torso must be accepted. All patients understand that the boomerang pattern originated with this author and that combining that operation with an abdominoplasty and posterior excisions is an exceptionally lengthy surgery. The operations are staged for BMI over 34, excessive skin resections, chronic illness, or patient concern. Patients agreed to be reported anonymously with consent obtained for photograph presentation.

**Fig. 3:**
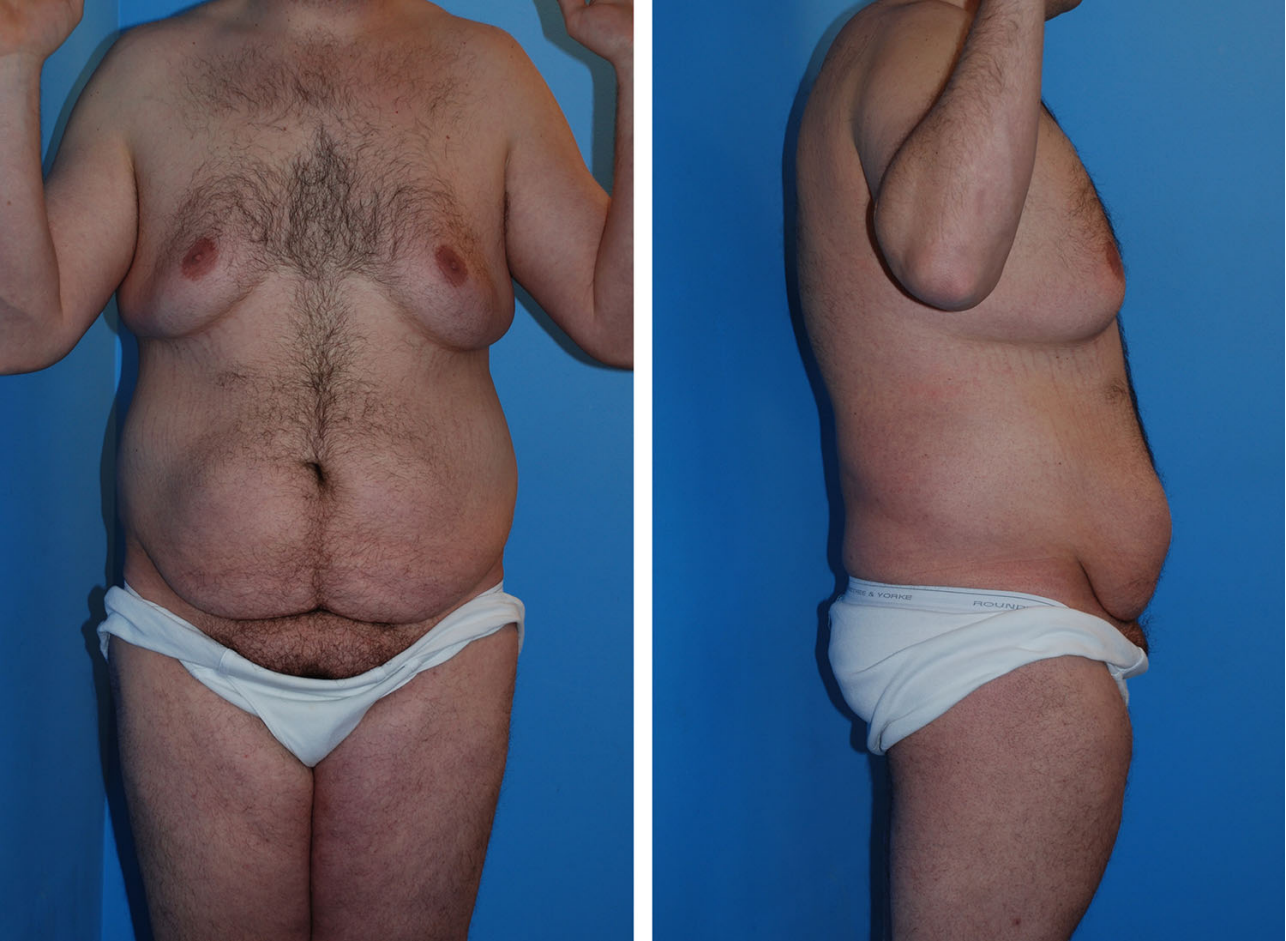
Frontal, right lateral, and posterior views of a typical massive weight loss male, Case 1, seeking comprehensive body contouring surgery to enhance muscular features. There is generalized mild to moderate adiposity with localized bulging and sagging of pseudogynecomastia, lower abdomen, flanks, mons pubis, and medial thighs

**Fig. 4:**
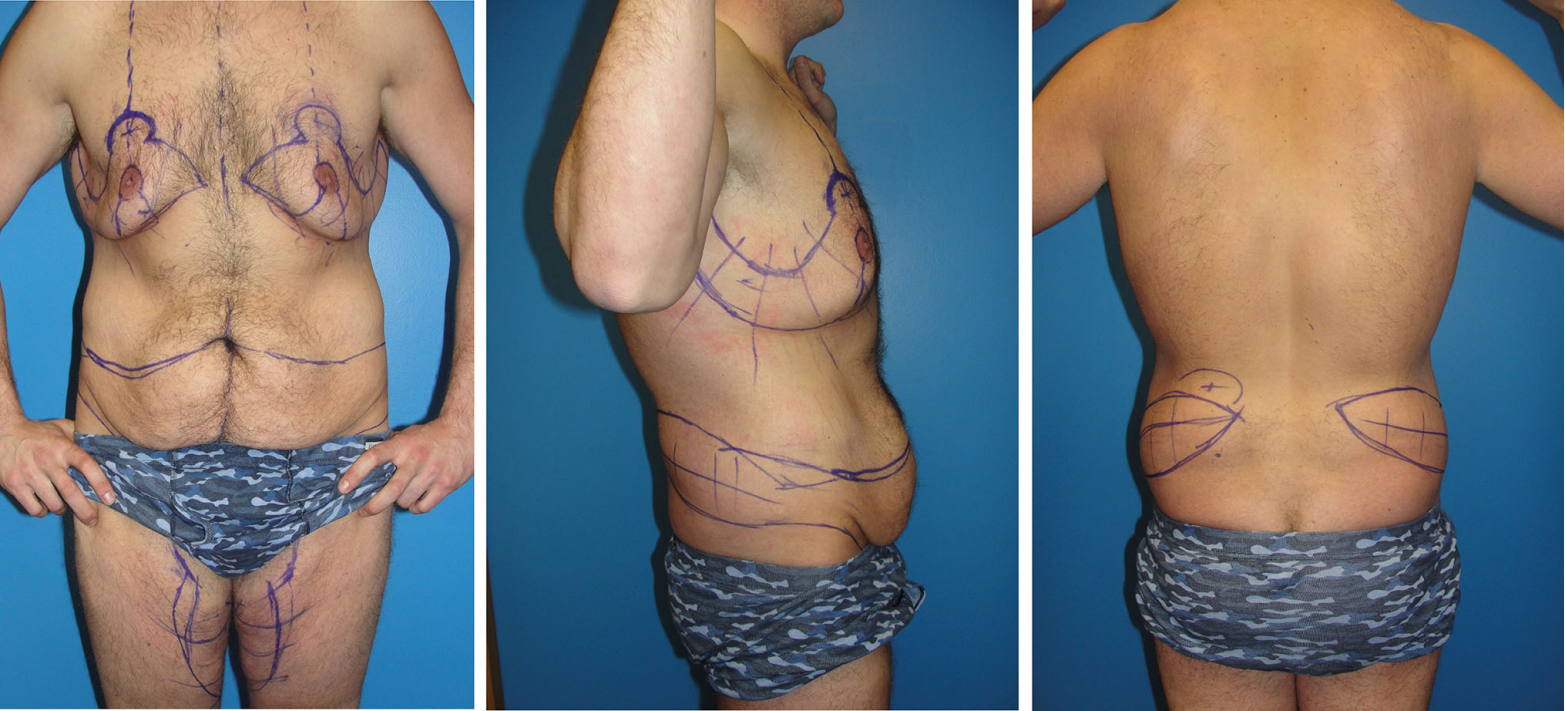
Frontal, right lateral, and posterior views after an additional 40 pound weight loss in Case 1 show he transformed to an ideal candidate for single-stage total body lift surgery. There is increased ptosis and definition of his gynecomastia, abdominal pannus, flank bulges, mons pubis, and medial thighs. Now the remaining non-resected skin with thin subcutaneous tissue could reveal superficial muscularity and upper body dominance. He has been marked for a boomerang pattern correction of gynecomastia, J torsoplasty upper body lift, and a central high tension abdominoplasty extended with elliptical oblique excisions of his bulging flanks and a picture frame pubic monsplasty extension of a vertical medial thighplasty

Case 1 (Table 1, patient 1) has generalized loose skin and residual adipose bulges of pseudogynecomastia, and lower body bulging abdominal pannus, mons pubis, and love handles (Fig. 3). This 27-year-old male lost 60 pounds, and regained 20 when frustrated by lack of muscular show. Through a 12-week HCG/500 calorie a day diet [5], he lost 40 pounds. The off label protocol starts with 100 units of subcutaneous Pregnyl (Merk.com) daily, which is adjusted to hunger and weight loss. At a reduced 28.2 BMI, his bulging breasts, abdomen, and flanks decreased in size and increased in laxity. One month after the HCG diet, he was marked for boomerang pattern correction of gynecomastia [6] and J torsoplasty [7] with a central high tension abdominoplasty extended by elliptical excisions of the flanks (Fig. 4). A vertical medial thighplasty combines with the abdominoplasty for a picture frame pubic monsplasty [8].

**Fig. 5:**
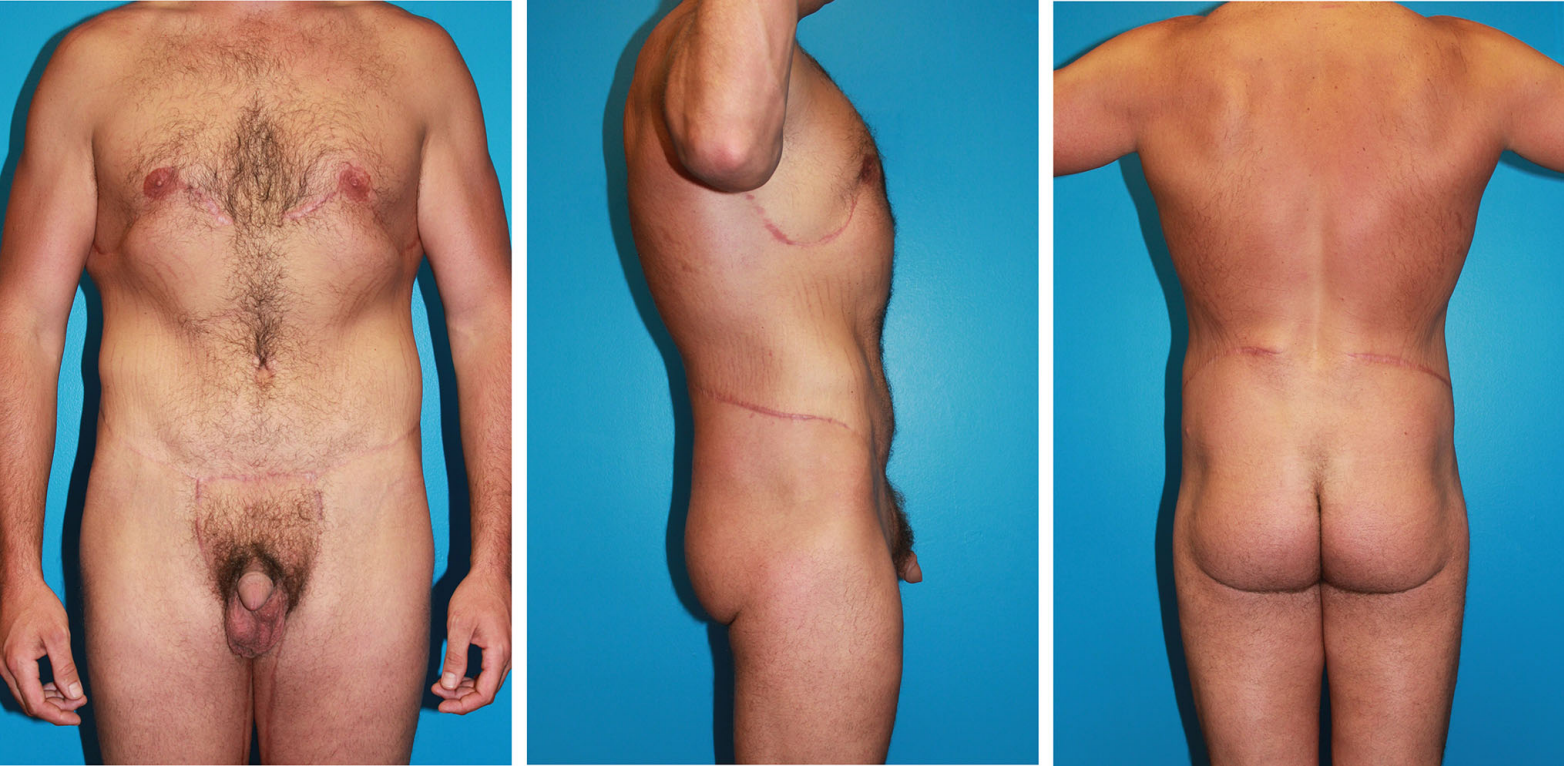
Case 1 improved contours and upper body dominance is evident 2 years and a body building extra ten pounds later. The shoulders including the deltoid masses are now broader than the waist and hips. A broad rib cage is draped with sheets of contoured muscles that taper to a narrow waist bordered by a flat abdomen and narrow rounded buttocks. A thin subcutaneous layer and adherent skin distinctly reveal the contour and edges of the pectoralis, latissimus, rectus abdominus, and external oblique muscles. Obliteration of pseudogynecomastia is combined with tightening of the upper torso skin. Upper body dominance is restored by effective reduction of lower torso excess

**Fig. 6:**
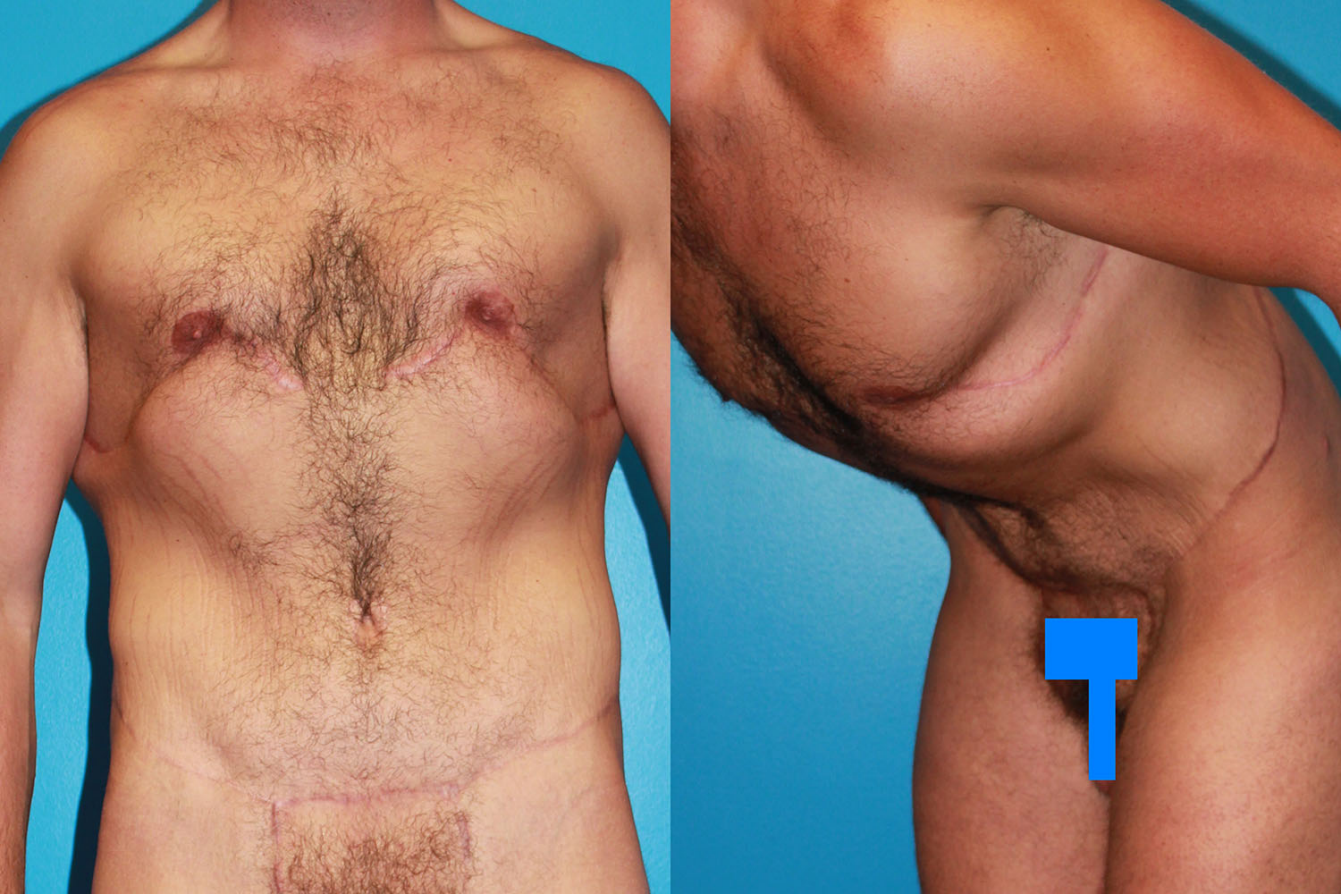
Frontal and left oblique swimmers view show that pectoralis major muscle contraction, relaxation, and arm and body position distinctively alter anterior torso surface form. *Left* Pectoralis muscle contraction bulges superior to the nipple and flattens inferior, even with the arm to the side. The defined inferior limit is the lowest origin of the pectoralis major muscle, not the IMF which is seen closer to the costal margin. *Right* Generalized even fullness is present behind the nipple with the pectoralis muscle relaxed and the arms to the side

Two years later, Case 1 has tight skin with visible muscularity and fading scars (Fig. 5). With arms elevated or upon muscular contraction, the pectoralis muscle bulges superiorly to the nipple and flattens inferiorly (Fig. 6, *left*). The diving position best shows tight anterior torso skin (Fig. 6, *right*). With the arms to the side and the pectoralis major relaxed, the skin about the NAC is filled with the superficial muscle, not breast.

## Methods, Materials, and Operative Technique

Office records review between January 2001 and March 2013 yielded 58 cases of excisional gynecomastia. There were 19 patients with the boomerang pattern, and abdominoplasty with posterior extensions by either lower body lift or oblique excisions over bulging flanks (Table 1).

The patient needs to be physically fit with good nutrition as determined by history, physical exam, and screening laboratory tests (CBC, BUN, electrolytes, Fe, coagulation profile and liver functions). Gastric bypass and malnourished patients were additionally screened for vitamin levels, trace minerals, and albumin. Surgical formula supplements including l-arginine (4 g) and l-glutamine (1 g) were provided through daily scoops of ProCare MD (Nutressential.com) mixed in a cup of liquid. Additional supplements were provided as needed. A ten-question non-validated satisfaction survey was designed and administered (Table 2).

**Table 2:** Patient survey muscular male

1. I am pleased with the overall result
2. I am comfortable shirtless in public
3. The gynecomastia is corrected
4. The chest scars are acceptable
5. The surgery improved the visibility of your upper body muscles
6. I need minor revision chest surgery
7. I need major revision chest surger
8. Lower abdominal skin excess is corrected
9. The abdominoplasty scar is acceptable
10. Love handles are corrected

(1) Disagree, (2) slightly disagree, (3) agree, and (4) totally agree

**Fig. 7:**
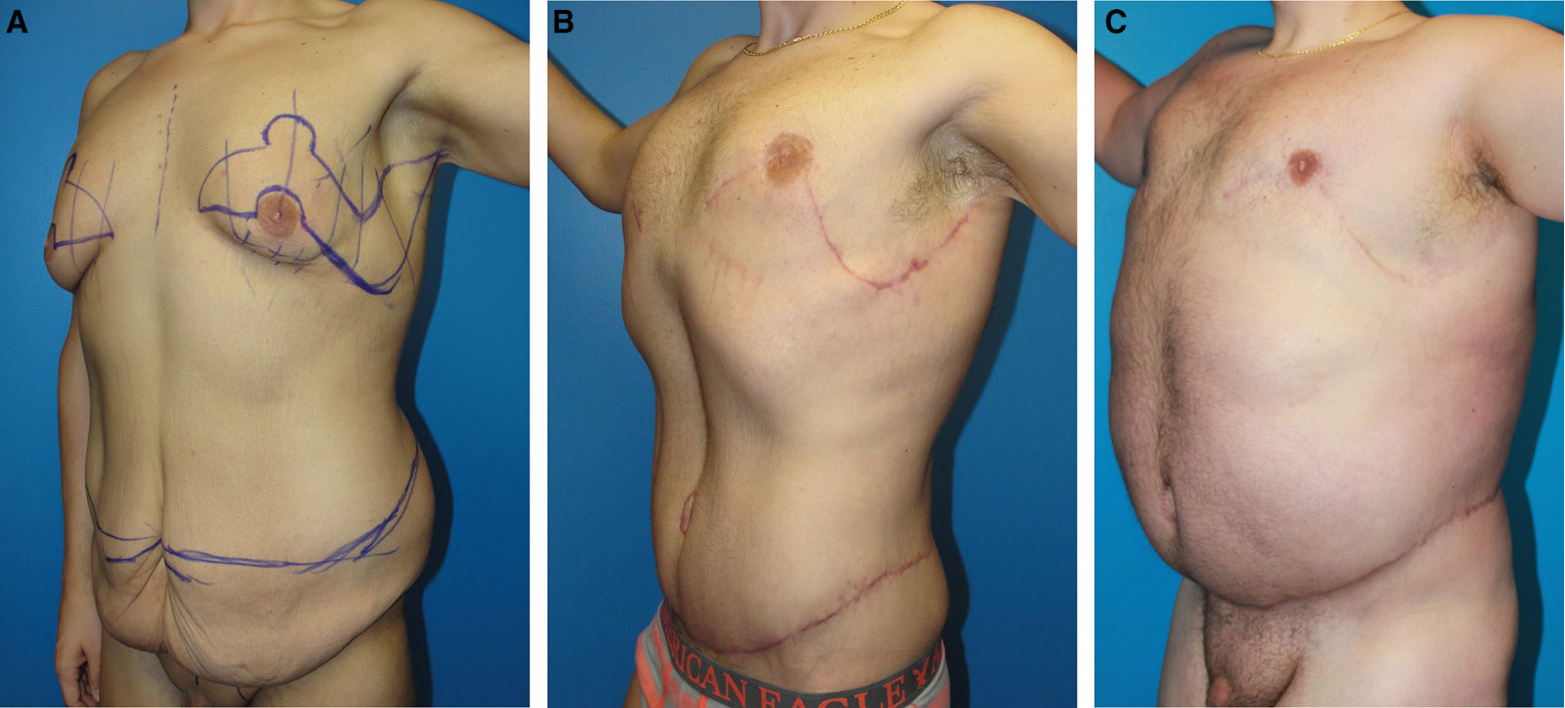
Case 2 left anterior oblique views before (**a**), and after TBL at 3 months (**b**) and 3.6 years (**c**). This 26-year-old, 6′1″ male requests body contouring surgery. After dieting from 390 to 200 pounds, he is embarrassed by gynecomastia, generalized loose skin, large sagging abdomen, and oversized love handles. **a** The *markings* are for boomerang pattern correction of gynecomastia with J torsoplasty extensions and abdominoplasty with oblique excisions over the flanks. **b** Three months later, the torso exhibits uniformly tight skin with oblique curvilinear scars seemingly interrupted along the superior margin of the NAC’s. The absence of lower torso bulge restores upper body dominance. **c** Three and a half years after his TBL, the scars have faded. Despite a 90 pound weight gain, the gynecomastia remains corrected. The abdomen is enlarged without ptosis or flank bulges

**Fig. 8:**
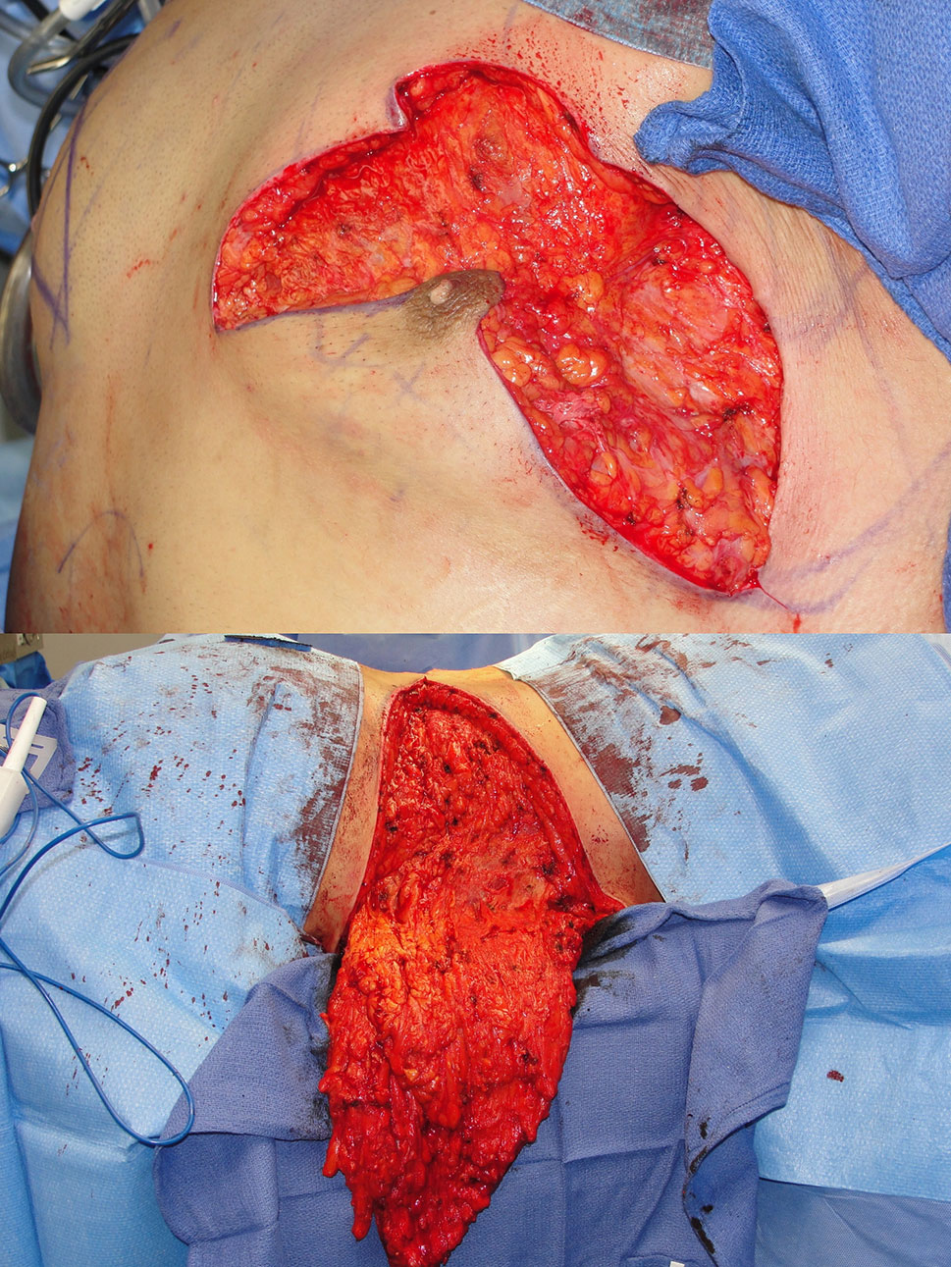
Case 2 intraoperative views of excisions. *Upper* After removal of the boomerang pattern, pectoralis muscle fascia is exposed. The inferior pedicle has been defatted and undermined through UAL. After this boomerang cutout is closed, the J torsoplasty pattern will be adjusted and excised to optimal width of resection. *Lower* Prone with head to the right. The dissected flank roll hangs from the yet to be resected abdominoplasty. The depth of resection is progressive to external oblique fascia

**Fig. 9:**
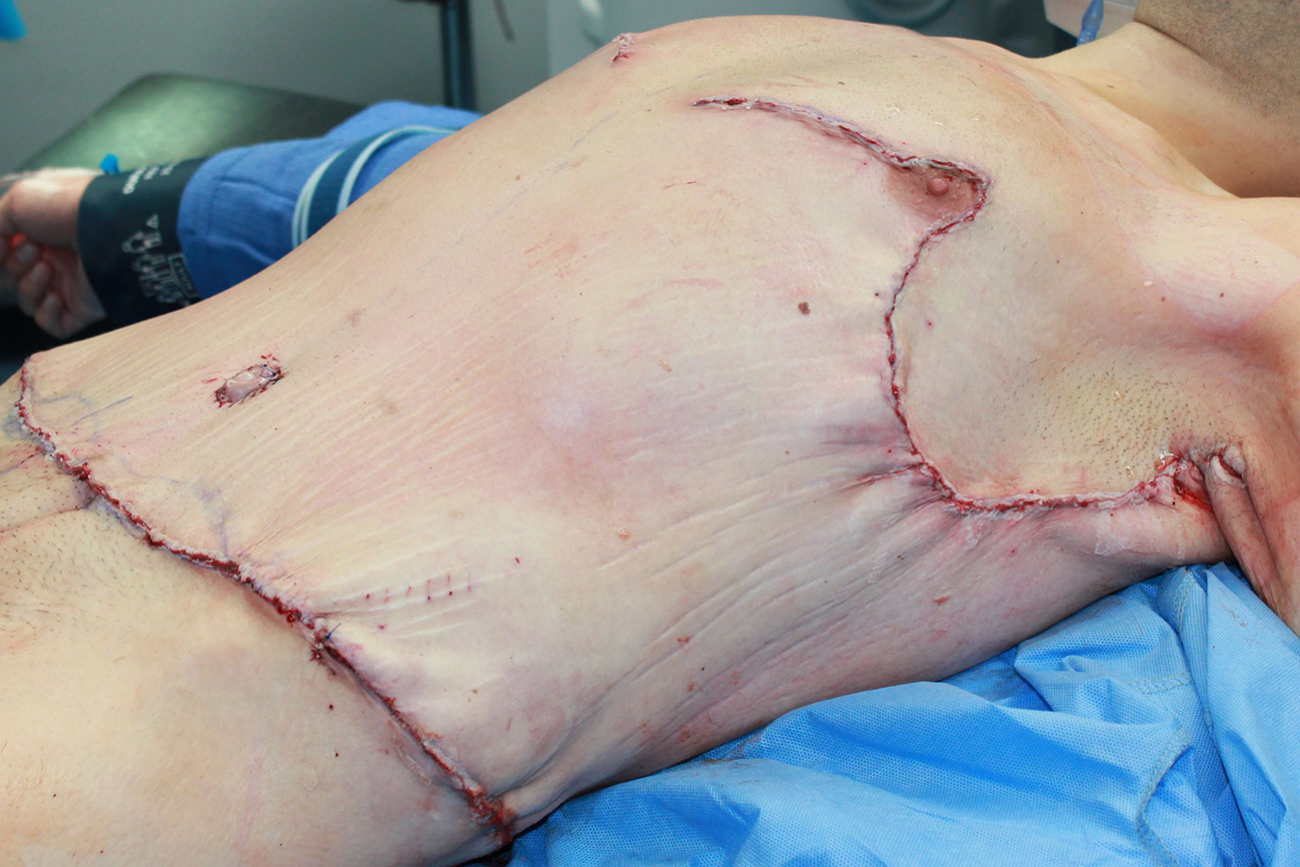
At the completion of the TBL, Case 2 shows uniform tension and improved contours across the anterior and lateral torso. The even pleating of the outer, longer incision line of the J torsoplasty smooths out over 3 months

Case 2 (Table 1, patient 13) lost 200 pounds prior to TBL and thighplasty markings (Fig. 7a). At 3 months, his TBL replaces bulging and loose skin for a tight wrap that reveals underlying muscles (Fig. 7b). By virtue of his reduced lower torso, his upper now dominates. Despite a disappointing 90-pound weight gain over the next 3 years, he retains masculine proportions with no gynecomastia or bulging lower torso (Fig. 7c). His intraoperative prone followed by supine positioning facilitates symmetry and two-team surgery. The flank excisions were medial to lateral progressively deeper resections. (Figure 8, *lower*). Two layer closure starts with running subcutaneous #2 PDO Quill on 48 mm. tapered half circle 72-cm-long *barbed* suture, and ends with intradermal 2-0 barbed Monoderm [9]. He was turned supine for abdominoplasty followed by upper body surgery. As the abdominoplasty is being sutured by the second team, the boomerang excision pattern including gynecomastia was excised over the pectoralis fascia [6] (Fig. 8, *upper*). After a two-layer Quill closure, the surgeon confirms the width of lateral vertical chest excision for the J torsoplasty. Upon completion of the TBL, improved contours are taut from clavicles to upper thighs with obliteration of the IMF (Fig. 9).

**Fig. 10:**
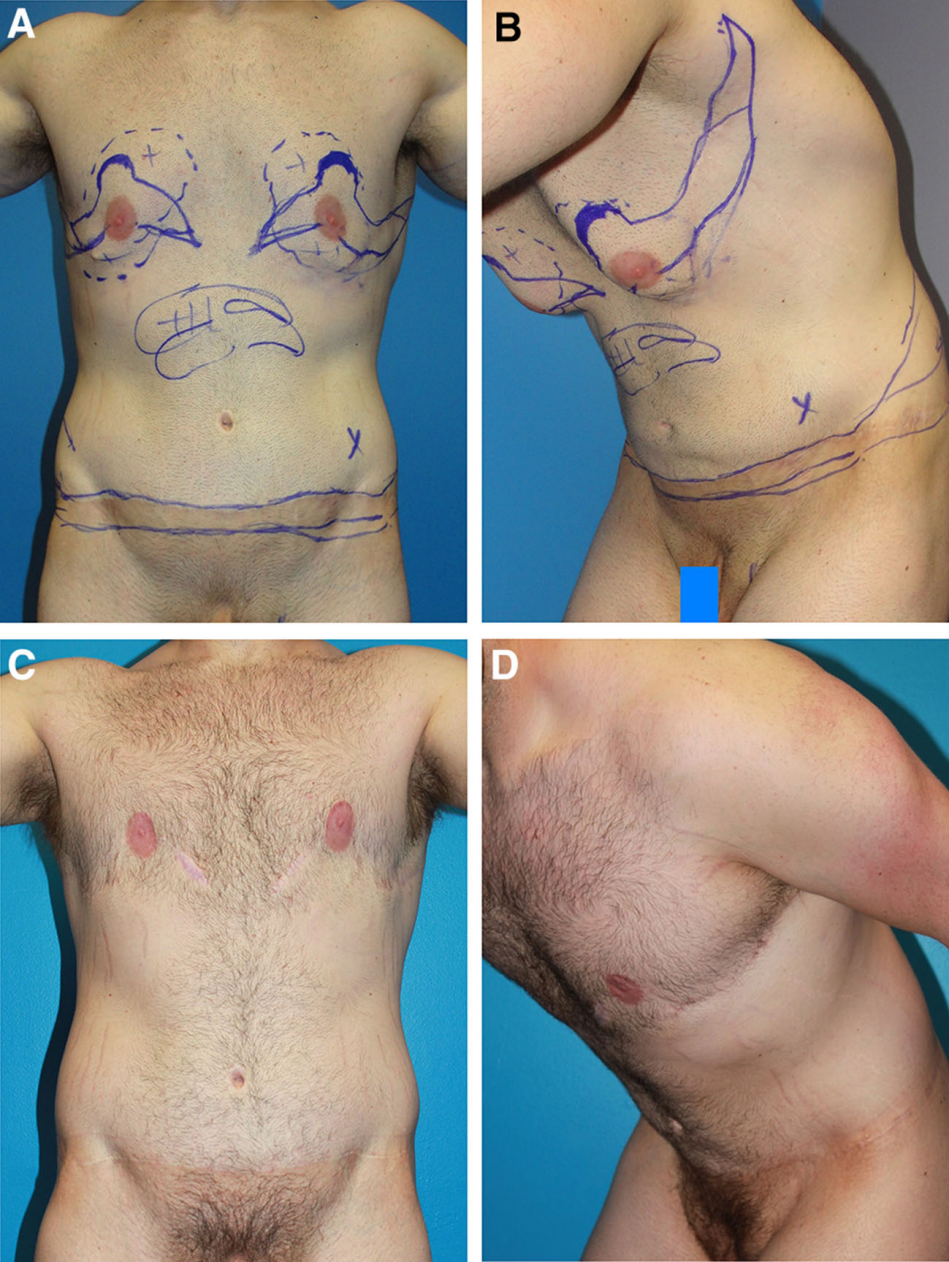
Case 3 is a 29-year-old with serial ellipses marked for revision surgery. He is 5′11″, 200 pounds, after 115 pound dietary weight loss. He is disappointed by gynecomastia, low nipples, and loose chest skin a year after two-stage body contouring elsewhere (**a**, **b**). The prior first stage was an abdominoplasty and lower body lift. The second stage was a transverse excision along the inframammary fold, extended to the lateral chest. His IMF remains defined, with no definition of pectoral borders. He was annoyed by anterior chest skin sagging when leaning forward, residual epigastric adiposity, high abdominoplasty scar, and left lower abdominal painful neuroma (*X*). The revision lines of resection had to be adjusted to include scars left by the original procedures. UAL of the upper torso is marked. **c**, **d** Two years following the revision, he has smooth, even contours, tight skin of the anterior torso with the torso appearing longer due to the greater distance between the lower abdominal scar and the raised NAC’s. His raised arms stretches the pectoralis muscles, which flattens inferior to the NAC’s. The oblique extension of his abdominoplasty revision further narrowed his waist. The lateral and inferior borders of the pectoralis major are defined, while the IMF’s are obliterated. The tightness of his chest skin is reflected by the absence of skin sag and prominence of his Pectoralis Major when leaning forward. The scars have faded

Case 3 (Table 1, patient 12) is marked for revision surgery (from elsewhere) consisting of boomerang pattern correction of gynecomastia with a J torsoplasty; liposuction of the epigastrium and anterior chest; tightening of the abdominoplasty with lowering the abdominoplasty scar; further flank resections; and muscle implantation of painful neuroma (Fig. 10a, b). The operations proceed as described for Case 2. Two years later, this combination of secondary operations satisfied this disgruntled patient (Fig. 10c, d).

## Results

The average age of the 19 patients was 36.5 (23–66). The average BMI at the time of TBL surgery was 28.5 (23–34.4). The average change in BMI was a 13.2 (2–25.8) reduction. The average follow-up was 3.1 (2–10.1) years. Aside from a high BMI, there were no comorbidities such as hypertension, diabetes, chronic pulmonary, cardiac, gastrointestinal, musculoskeletal, or neurologic diseases.

Seventeen cases were single-stage TBL. Six of the first eight had transverse upper body lift extensions of their boomerang correction. The last 11 patients underwent J torsoplasty extensions. Eight of the first nine patients had transverse lower body lift extensions of their abdominoplasty. Nine of the last ten patients had oblique excision extensions directly over the flanks.

The last eight single-stage TBL operative sessions with oblique flank excisions (Patient #10-17) averaged 4 h and 20 min (range 3′24″–6′17″). Time of surgery seemed directly related to patient BMI and height, plastic surgery resident inexperience, and thighplasty and brachioplasty. Detailed photographic or video documentation increased operative time. Two-layer running barbed suture closures reduced time.

All patients were discharged after an overnight hospital stay. At 1 week suction, drains and sutures were removed. Single-stage TBL patients returned to usual activities within 6 weeks. Two-stage patients needed half that time.

The author contacted and received a response to the survey (Table 3) from all 19 patients. Two patients were not completely satisfied with the improvement in their chest. Case 2 did not have a posterior extension to his boomerang excision, which left him with mid-lateral chest bulges. Case 15 requests secondary liposuction of his inferior pedicle. Only one of the first eight patients with transverse lower body lift were completely satisfied with the correction of their love handles; whereas, all of the last nine cases with direct oblique excisions were satisfied.

**Table 3:** Patient survey

**#**	Q1	Q2	Q3	Q4	Q5	Q6	Q7	Q8	Q9	Q10
1	3	3	4	4	3	1	1	4	4	2
2	3	3	3	2	3	4	1	4	4	2
3	4	3	4	3	4	1	1	4	4	2
4	3	4	4	2	4	1	1	3	4	2
5	3	4	3	3	4	4	1	4	4	3
6	3	4	4	4	4	1	1	4	4	1
8	4	4	4	4	4	1	1	4	4	4
10	3	4	4	4	3	1	1	3	4	2
11	4	4	4	4	4	1	1	4	4	4
12	4	4	4	4	4	1	1	4	4	3
13	4	4	4	4	4	1	1	4	4	4
14	4	2	4	2	4	3	1	4	4	3
15	4	3	2	3	4	3	1	4	4	3
16	4	4	4	4	4	1	1	4	4	4
17	4	4	4	4	4	1	1	4	4	4
18	4	4	4	4	4	1	1	4	4	4
19	4	4	4	4	4	1	1	4	4	4

Five patients had significant complications (additional treatment and/or prolonged recovery) for a rate of 26 % of the patients (Table 1). There was an average of 4.3 major procedures per patient. Hence, for the total of 74 procedures, there was a 6.7 % complication rate per procedure. Patient #3 required drainage of a hip seroma. Patient #4 suffered distal necrosis of the fleur de lis (FDL) abdominoplasty, which was debrided followed by secondary closure. Patient #5 had distal superior NAC necrosis that healed secondarily. Patient #6 required operative drainage of a chest hematoma and a two-unit transfusion. Eight years later, #6 underwent liposuction of his flanks with lipoaugmentation of the lateral gluteal depressions with partial success. Patient #7 required drainage of a buttock seroma and decortication of a buried deepithelialized flap. Patient #9 did not have a wound-healing complication, but he complained of superior placement of the nipple areolar complexes. Repositioning caused NAC vertical elongation.

**Fig. 11:**
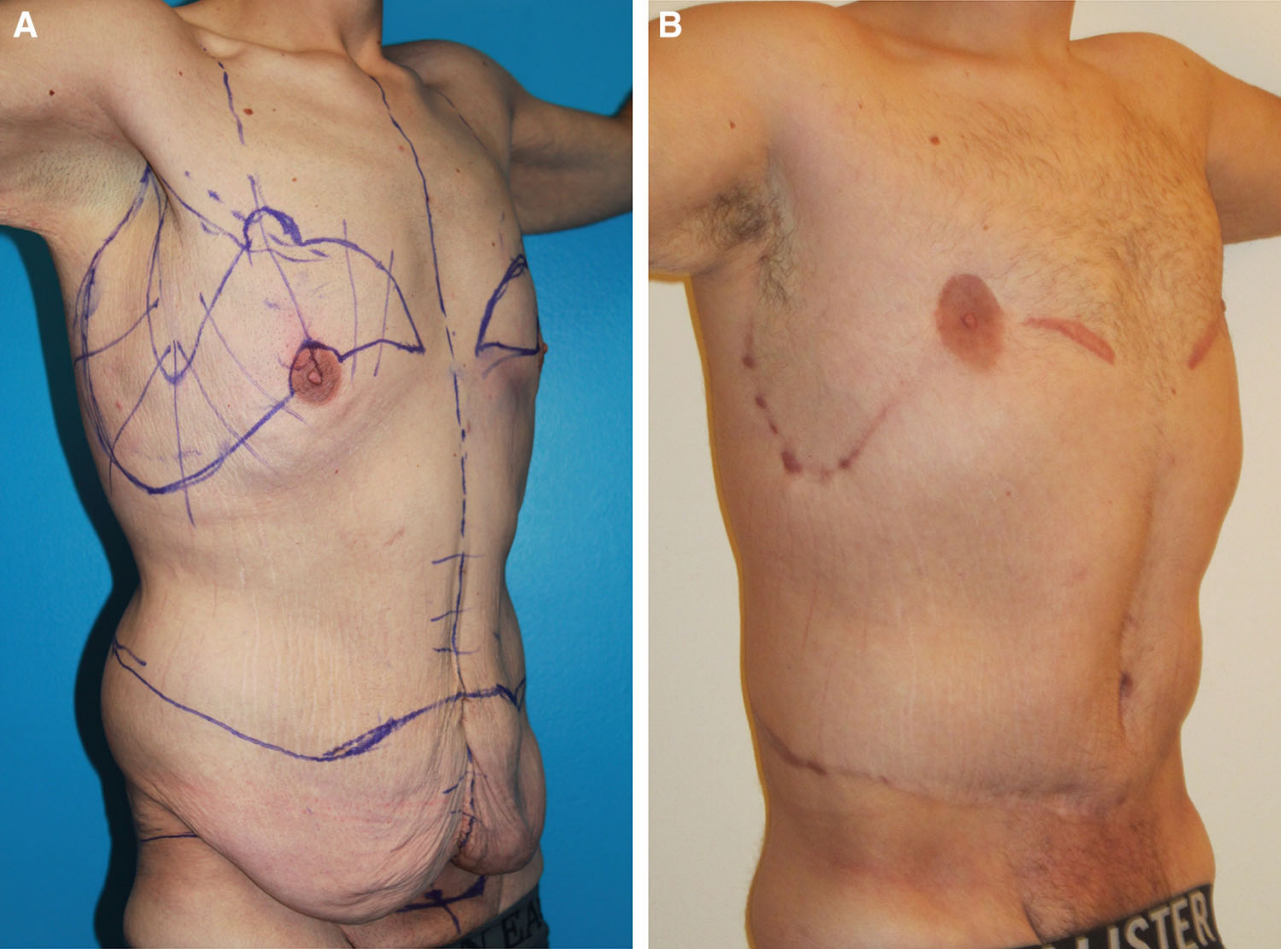
Case 4 show hypertrophic medial chest scars in a 26-year-old, who lost 210 pounds to a BMI of 23.6. **a** Right anterior oblique view with preoperative markings for boomerang pattern correction of gynecomastia with J torsoplasty, and abdominoplasty with oblique excisions over the flanks. **b** Same view nearly 3 years later shows tight skin revealing muscular contours and dominance of the upper torso. Although improved, hypertrophy of the medial chest scars has not resolved

 The superior margin of three NAC’s suffered minor superficial skin necrosis and all healed secondarily with no deformity. Patient #14 has not resolved his hypertrophic medial chest scars after nearly 3 years (Fig. 11). While pleased with his overall contours, muscular show, and upper torso dominance, he is self-conscious when he exposes his chest on the beach. Case 15 is considering liposuction of residual inferior pedicle chest fat. One patient gained 30 and another patient gained 90 pounds.

## Discussion

Loose skin and gynecomastia were corrected in all 19 patients. Nine of the last 11 patients avoided a back scar with a J- torsoplasty and completely lost their bulging flanks through direct oblique excisions. Oblique flank excisions left no lateral gluteal depressions.

The boomerang and J torsoplasty is for moderate to severe chest skin laxity. Isolated gynecomastia is treated with liposuction and glandular pull through resection through a periareolar incision and possible mastopexy [10]. Extreme skin laxity with gynecomastia cannot be corrected by transposing the NAC a relatively short boomerang pattern distance [11].

Obliquely oriented closures over the anterior chest are aesthetic for gynecomastia correction [12]. By encircling the superior NAC, the long anterior chest is aesthetically interrupted. Continuation through a lateral torsoplasty hides that scar under the resting arm. Medial chest scar hypertrophy occurs rarely and takes years to resolve. Mid-chest transverse excisions remove most vertical but little horizontal excess, leaving sagging skin to obscure muscular detail (Case 3). A long transverse excision, that may have to cross the sternum, reestablishes an undesirable IMF. An inferior pedicle buried deepithelialized flap to the NAC leaves disturbing inferior fullness.

Five of the first seven patients in this 19 case series required treatment of complications. One lateral chest hematoma was evacuated. This is consistent with the reported higher incidence of hematoma (14.6 %) and seroma (25 %) in men having body contouring surgery after MWL [1]. Multiple operations are common, as Rubin’s group noted 59.6 % of the 48 male patients had more than one procedure.

Success in these combinations of operations fulfills the concept that both horizontal and vertical skin excess can be removed through a series of complimentary angled long oblique excisions [3]. If the residual skin is anticipated to carry too much adipose then the surgery should be delayed until further generalized weight has been lost (see Case 1).

Since men represent only about 10 % of body contouring surgery, [1] plastic surgeons understandably, but erroneously apply techniques to men designed for women. Inferior buried pedicle flaps for correction of pseudogynecomastia [11] and bikini level lower body lift incisions [13] are two examples.

To date, the goals of liposuction of the muscular male torso have been more artistic than for excisional body contouring surgery. Mentz introduced differential liposuction for etching of the male torso, especially in the poorly defined weight lifter [14]. Observing male aesthetics, Hoyos has elevated liposculpture to 3-dimensional VASERlipo of subcutaneous adipose with lipoaugmentation of superficial muscles. [15] In non-MWL men, Rizzo “muscular sculpts” through lipoplasty. After MWL, men undergo a preliminary abdominoplasty with gynecomastia removal and periareolar mastopexy [16].

Regarding excisional surgery, most reports of male body contouring surgery are limited to treatment of gynecomastia. In a review of 48 male MWL contouring body patients, Rubin’s group focuses on gender variances in preoperative presentation and post-operative complications, not gender aesthetics [1]. Their males presented with a greater change in BMI and less clinical depression than women. The rate of 42 % overall complications, mostly hematomas and seromas, was statistically greater than females.

Recently, Rubin edited a 14-article review on body contouring, in which he presented one male case [17]. Capella reserves the combination of abdominoplasty and lower body lifts with vertical medial thighplasty for android appearing women and males [18]. Richter and Stoff wrote, “Male patients frequently present with local adipose tissue in the posterior flank region, which may be reduced by liposuction after gluteal wound closure, or superiorly excised in toto during posterior preparation.” [19] This author has found the later choice more effective.

## Conclusion

Boomerang excision pattern correction of gynecomastia and J torsoplasty are combined with an abdominoplasty with oblique excisions directly over bulging flanks for effective and safe optimizing of muscularity and upper torso dominance in the MWL male. Further clinical study will determine the reliability and safety of this approach. More cases and technical details are forthcoming [20].
